# Mast cells infiltrates are common in eosinophilic esophagitis and still elevated in histological remission: A digital evaluation in children

**DOI:** 10.1002/jpn3.70137

**Published:** 2025-07-02

**Authors:** Theresa Hironimus, Sabrina Degen, Anja Rappl, Ida Allabauer, Joachim Woelfle, Arndt Hartmann, Volker Bruns, Rosalie Kletzander, Carol Geppert, André Hoerning

**Affiliations:** ^1^ Department of Pediatrics University Hospital Erlangen, Friedrich‐Alexander‐Universität Erlangen‐Nürnberg Erlangen Germany; ^2^ Institute of Medical Informatics, Biometry and Epidemiology, Friedrich‐Alexander‐Universität Erlangen‐Nürnberg Erlangen Germany; ^3^ Institute of Pathology, University Hospital Erlangen, Friedrich‐Alexander‐Universität Erlangen‐Nürnberg Erlangen Germany; ^4^ Comprehensive Cancer Center Erlangen‐EMN (CCC ER‐EMN), University Hospital Erlangen, Friedrich‐Alexander‐Universität Erlangen‐Nürnberg Erlangen Germany; ^5^ Fraunhofer IIS Erlangen Germany

**Keywords:** allergy, CD‐117, Congo Red, digital image analysis, pediatric

## Abstract

**Objectives:**

Eosinophilic esophagitis (EoE) is a type 2 inflammatory chronic allergic disease with several additional immune cell subsets being involved. The aim of this study was to assess the identification and quantification of mast cells (MC) infiltrates using an objective and examiner independent analysis via digital image analysis of digitized histochemically stained biopsies.

**Methods:**

Biopsies were taken from the esophagus of 24 children and adolescents diagnosed with EoE and stained for MC and eosinophilic granulocytes using anti‐CD117 and Congo red, respectively. Samples were digitized, eosinophilic granulocytes and MCs were quantified using the MIKAIA® image analysis software.

**Results:**

At diagnosis MC infiltrations were regularly observed in active disease. MC numbers were 160 (cells/mm^2^) before therapy initiation and 33 (cells/mm^2^) in histological remission (<15 Eos/HPF). The number of mucosal MCs at the time of remission decreased less than that of eosinophilic granulocytes, regardless of the initiated therapy. Therefore, patients in histological remission with eosinophilic granulocytes showing on average 2,4 cells/mm^2^ still exhibited MC infiltrations (average 33 cells/mm^2^). Furthermore, male patients displayed higher numbers of eosinophilic granulocytes at time of diagnosis. With regard to the site of biopsy sampling, an accumulation of the MC count in the distal direction can be observed.

**Conclusions:**

Mast cells are involved in EoE and persist after achieving histological remission. Both CD117 + MC and eosinophilic granulocytes can be quantified using MIKAIA® as a tool for objectifying the histological diagnosis of EoE.

**Trial Registration:**

Identification number: DRKS‐ID 00014688; https://drks.de/search/de/trial/DRKS00014688

## Abstract (highlights)

**What is Known:**

- Mucosal mast cell infiltrations have been described in active eosinophilic esophagitis (EoE) and seem to play a role in pathophysiology.
- Digital histological diagnosis of EoE is an unmet need.

**What is New:**

- Mast cell (MC) infiltration persist although histological remission per standard criteria (<15 Eos/HPF) is achieved.
- Higher number of esophageal eosinophilic granulocytes in male patients at the time of diagnosis.
- Differences of MC counts in esophageal segments with accumulation in the mid and distal localizations.
- Both CD117+ mast cells and eosinophilic granulocytes can be quantified using MIKAIA® not only objectifying but also alleviating the histological diagnosis of EoE.

## Graphical abstract

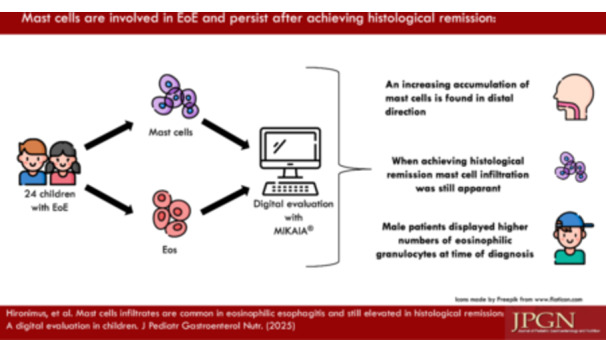

## 1 INTRODUCTION

Eosinophilic esophagitis (EoE) is a chronic, allergic inflammatory disease with an increasing incidence and prevalence.^1^ It is mainly characterized by esophageal dysfunction.^2^, ^3^ The gold standard of diagnosis is assessing the eosinophilic granulocyte infiltration at the proximal, middle and distal esophageal segments.^3^ While in childhood EoE has mainly an inflammatory character, adults usually show a more fibrotic stage of the disease.^1^, ^4^ In general, a dynamic course of the type 2 inflammation can be observed, which may lead to fibrosis even in children with strictures that predominate distally.^5^

It is known that EoE is caused and maintained by various factors, including environmental factors (especially allergens), as well as various genetic factors play an important role. For example, first‐degree relatives have an increased risk of developing EoE^6^ and a predominance in males has also been identified.^7^, ^8^ In addition, the identification of disease specific genes, such as thymic stromal lymphopoietin, kallikrein, anoctamin 1 and calpain 14 has been linked to the development of the disease.^3^, ^6^

Regarding the composition of immune cellular infiltrates that contribute to and perturbate the development as well as allowing the maintenance of the disease, mast cells (MC) may play an additional role.^9^ MC are important amplifiers involved in several types of immune reactions and are found in varying frequencies in almost all tissue types. They are particularly abundant at interfaces where the body encounters the environment such as the mucosal surface of the gastrointestinal tract or the respiratory tract. When activated, these granules‐filled cells cause the release of immunomodulatory compounds, which can have both protective and damaging effects.^10^ To the best of our knowledge, there are no studies on the cell count of MC and eosinophilic granulocytes in relation to the esophageal segments and therapy response in pediatric patients. Furthermore, we focused on digital evaluation of cellular infiltrates as this emerges to be an alternative to manually counting cells in the near future and this procedure improves not only the objectivity but also the pathologist's workload.

## 2 METHODS

### 2.1 Ethics statement

This is a retrospective analysis of a monocentric cohort, it was approved by the local ethics committee (#317_16B). All subjects provided informed consent before study inclusion.

### 2.2 Patients

Inclusion criteria for patients aged 0–18 years suffering from clinical signs of dysphagia or esophageal dysfunction corresponded to the current ESPGHAN/NASPGHAN guidelines^8^ in combination with pathologically increased esophageal tissue eosinophilia ( ≥ 15 eosinophils/high power field; 1 HPF = 0.25 mm²) in every esophageal segment (a total of 6–12 biopsies, preferably from visible lesions, obtained from the proximal, mid and distal levels of the esophagus). The histological inclusion criteria were deliberately chosen to be stricter than the current guideline recommendations for the purpose of investigating the mast cell infiltrations in every segment at baseline and in the longitudinal course.

Patients treated with proton pump inhibitors (PPI) after diagnosis of EOE received omeprazole 2 × 1mg/kg body weight with an upper limit of 40 mg*2 per day, therapy effects were investigated by collecting biopsies at proximal, mid and distal segments of the esophagus. Proton pump inhibitor (PPI) therapy refractory patients (>15 Eos/HPF at least in one of the three segments) were subsequently treated either with an empirical six food elimination diet (FED), or an exclusive elementary aminoacid based diet or a topic oral budesonide therapy (TCS) using a viscous solution under a normal unrestricted diet, each followed by an endoscopy to evaluate the therapy effect. The dosage of TCS was budesonide 2 × 0.5 mg per day for children < 10 years of age or 2 × 1mg per day ≥ 10 years of age. All patients were therapy naive, however, PPI therapy was not always first choice and in some patients it was implemented after an ineffective elimination diet such as 1FED (cow milk), or 6FED. Follow up endoscopy was performed 2–3 months after treatment initiation or treatment alteration regarding dosage or change in medication. The statistical analysis focused on biopsies at diagnosis of EoE and achievement of histological remission defined as tissue levels of eosinophilic granulocytes <15/HPF as assessed in all esophageal segments. Because of dosage adjustment in some patients the calculation of the time to histological remission especially for the PPI responding patients yielded a longer period of time as usually (Table 1).

**Table 1:** Overview of study participants.

	At diagnosis (*N* = 24)	At remission (*N* = 23)
Eosinophilic granulocytes^1^ (per mm²)		
Median [Q25, Q75]	245 [115, 365]	2.38 [0.955, 8.59]
Mast cells^1^ (per mm²)		
Median [Q25, Q75]	160 [93.6, 265]	32.7 [18.5, 42.8]
Treatment		
PPI nonresponders	16 (66.7%)	15 (65.2%)
PPI responders	8 (33.3%)	8 (34.8%)
Gender		
Male	15 (62.5%)	15 (65.2%)
Female	9 (37.5%)	8 (34.8%)
Weight (percentiles)		
Median [Q25, Q75]	21.5 [4.25, 56.0]	37.0 [7.50, 58.0]
Weight (categorized)		
Normal	18 (75.0%)	19 (82.6%)
Dystrophic	6 (25.0%)	4 (17.4%)
^1^(Average across all biopsies)		
Age at diagnosis (years)		
Median [Q25, Q75]	4.37 [1.13, 13.5]	
Time to remission (months)		
Median [Q25, Q75]	9.24 [4.15, 16.8]	
Time to first relapse (months)		
Median [Q25, Q75]	4.60 [2.84, 7.79]	
Time to first relapse (categorized)		
Fast relapse (<7.8 months [Q75])	11 (45.8%)	
Slow/no relapse (>=7.8 months [Q75])	12 (50.0%)	

Abbreviation: PPI, proton pump inhibitors.

### 2.3 Esophagus biopsy sampling and tissue staining

The biopsies were taken during esophagogastroduodenoscopy using special forceps with an outer diameter of 2.2 mm. These were taken from three segments in the esophagus and then placed directly in formalin (4% buffered). After the tissue was embedded in paraffin, sections were made at a thickness of 2 µm. The sections were mounted on glass slides and stained with hematoxylin‐eosin (HE) for diagnostic evaluation. New sections were prepared specifically for our study and stained with anti‐CD117 (c‐kit) and Congo red using an automated staining system (Benchmark, Ventana Medical Systems Inc., Tucson, AZ, USA). In contrast to the MC, the eosinophilic granulocytes were not visualized by immunohistochemical staining but by standard Congo red histochemical staining. For each esophageal segment (distal, mid, proximal), one out of four biopsies were processed for sectioning and subsequent staining for anti‐CD117 and Congo Red.

### 2.4 Digitization and detection

Slides with freshly stained sections were completely scanned and digitized (Panoramic P1000, 3DHistech, Budapest, Hungary; scanner software version 2.1.1., resolution 0.23 µ/pixel) and subsequently reviewed regarding quality. Quality Control (QC) and digitization issues (e.g., check for sharpness, tissue detection, region of interest [ROI] etc.) were checked using slides viewer software (SlideViewer 3DHistech, Budapest, Hungary; Version 2.7.0.). We used the MIKAIA® studio v1.5.1 software (RRID:SCR_025081, Fraunhofer IIS, Erlangen, Germany, www.mikaia.ai) to analyze the sections. The digitized slides were prepared and analyzed in the same way for all stains (Supporting Information: Figures 3 and 4). The detailed workflow of the analysis is presented separately (Supplemental Information: MIKAIA® S7).

### 2.5 Statistical analysis

Histological remission was defined as esophageal infiltration of eosinophilic granulocytes <15/HPF for all esophageal segments (distal, mid, and proximal). Levels of eosinophilic granulocytes and MC in pediatric individuals at the time of diagnosis and remission as well as the change of each cell type over this period were compared using adequate tests, that is, cell counts with a paired two‐sample Wilcoxon test (for non‐normal data) and the cell change as relative difference = (cell count after − cell count prior)/cell count prior with a paired two‐sample t‐test (for continuous, normal data). Further, subset cell counts and changes of eosinophilic granulocytes and MC were analyzed, and whether they differed significantly within specific subgroups, for example, therapy response, tissue type, esophageal segments, gender and slow or fast histological relapse. We used the data on each individual's progression after their remission to determine the time to first relapse. We then created the slow (or no) relapse (x < Q75) and fast relapse (x ≥ Q75) groups based on the 75% quantile of all observed times to first relapse (Q75) and adjusted for multiple testing within each subgroup using the Bonferroni method, but not between subgroups due to the exploratory nature of our analysis.

We further investigated whether the cell count and change of the specific attributes of the subgroups differ within each cell type and time point. Paired tests were used for subgroups tissue type and esophageal segments, all others constituted non‐paired samples. Again, cell counts were tested with the Wilcoxon test, cell changes with a t‐test. The subgroup esophageal segment with its three distinct attributes (distal, mid, proximal) was tested with the Friedman test and a repeated measures ANOVA. Significance level was *α* = 0.05, with a *p*‐value < 0.05 statistically significant. Relevant group comparisons are illustrated using boxplots.^11^

For the data analysis if the level of MC at remission is associated with specific clinical symptoms, we restricted to a descriptive analysis only due to the small sample size.

## 3 RESULTS

The patient cohort consisted of 24 children and adolescents (male *n* = 15, 62.5%) aged 0.5–17.5 years in the period from 2013 to 2022. Median age at diagnosis of EoE was 4.37 years (Q25‐Q75 1.13–13.5). From 24 children and adolescents a total of 137 slides were analyzed for anti‐CD117 and 135 slides for Congo red staining.

Median esophageal infiltration of eosinophilic granulocytes was 245 cells/mm² (Q25‐Q75 115–365), and 2.38 (Q25‐Q75 0.96–8.6) at the time of diagnosis and histological remission, respectively. The median esophageal infiltration of MC was 160 cells/mm² (Q25‐Q75 93.6–265) and 32.7 cells/mm² (Q25‐Q75 18.5–42.8), respectively. Eight patients achieved histological remission by PPI therapy (33.3%), the remaining PPI‐nonresponders (PPI‐NR) patients received other treatment options, such as 6FED (seven patients), elemental diet (two patients), or topical budesonide therapy (seven patients).

Patients presented with a median weight at the 21.5‐percentile at the time of diagnosis (Q25‐Q75 4.25–56.0) and about a quarter (six patients) were dystrophic. The median time to histological remission was 9.24 months (Q25‐Q75 4.15–16.8) and the median time to first relapse was 4.60 months (Q25‐Q75 2.84–7.79). With a threshold for fast relapse set at Q75 = 7.8 months the cohort consisted of 11 patients with fast relapse and 12 with slow or no relapse (Table 1 and Supporting Information: Figure 2).

**Figure 1:**
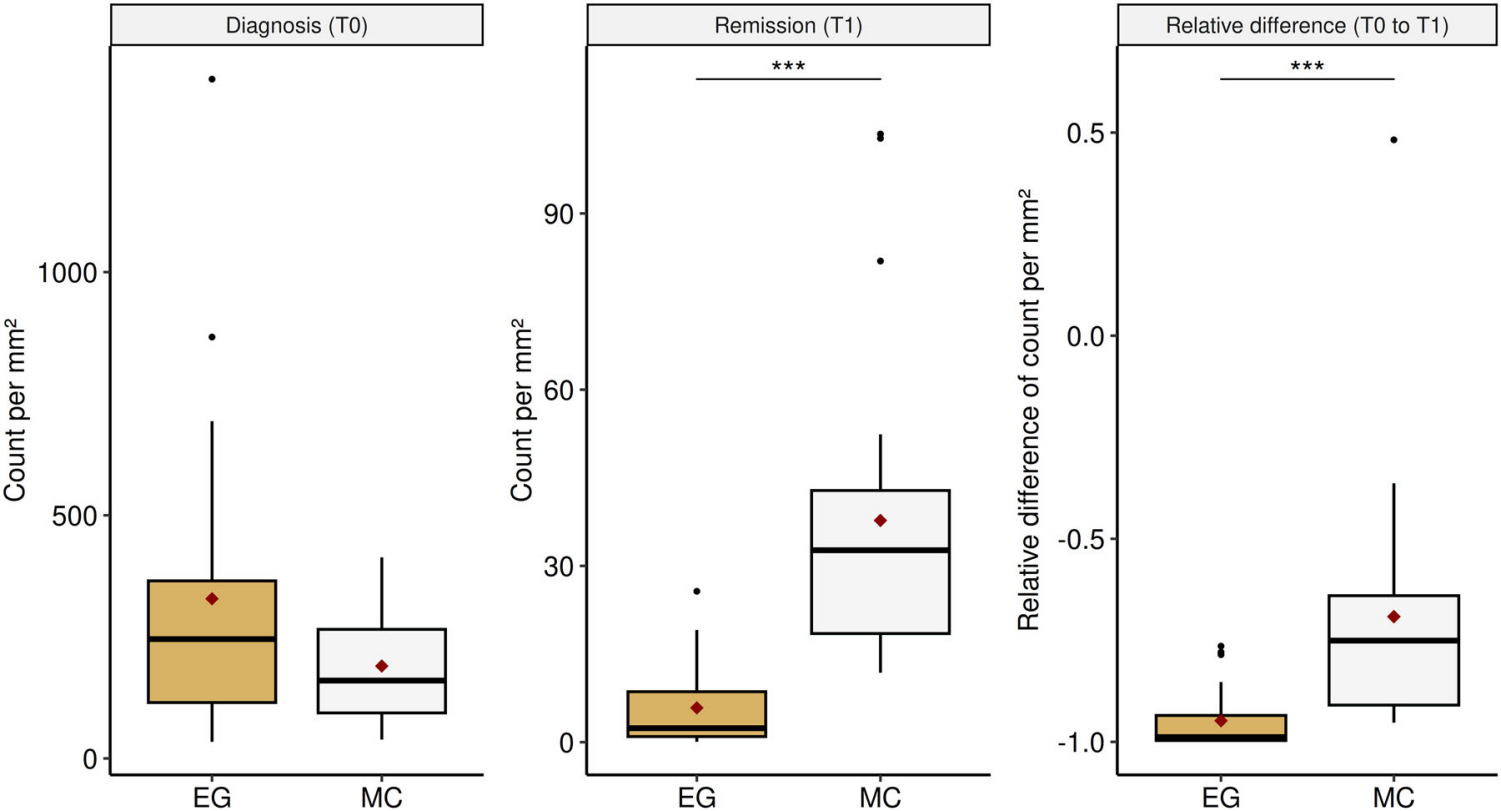
Cell counts of eosinophilic granulocytes (EG) and mast cells (MC) at diagnosis, at remission and cell changes during that period. The diamonds represent the mean of each group. *p*‐Value: <0.001***, <0.01**, <0.05*.

There was no significant difference of the esophageal counts of eosinophilic granulocytes (median 245.42 (Q25‐Q75 114.91–365.09)) and MC (median 160.08 (Q25‐Q75 93.61–265.38)) at diagnosis, however, there was at histological remission. The level of MC (median 32.67 (Q25‐Q75 18.49–42.84)) was significantly higher at this time point compared to the numbers of eosinophilic granulocytes (median 2.38 (Q25‐75 0.95–8.59), Figure 1 and Supporting Information: Table 1).

Further, we examined whether the level of eosinophilic granulocytes differed from the level of mast cells within specific subgroups at the two time points. The subgroups comprised type of therapy (PPI‐responders [PPI‐R] and PPI‐NR), tissue type (squamous epithelium, other tissue), esophageal segments (proximal, mid, and distal), gender (male and female) and relapse time (slow and fast). The results were all similar in the sense that no difference in cell type count could be detected at diagnosis but was significant for all subgroups at remission (Supporting Information: Table 1).

**Figure 2:**
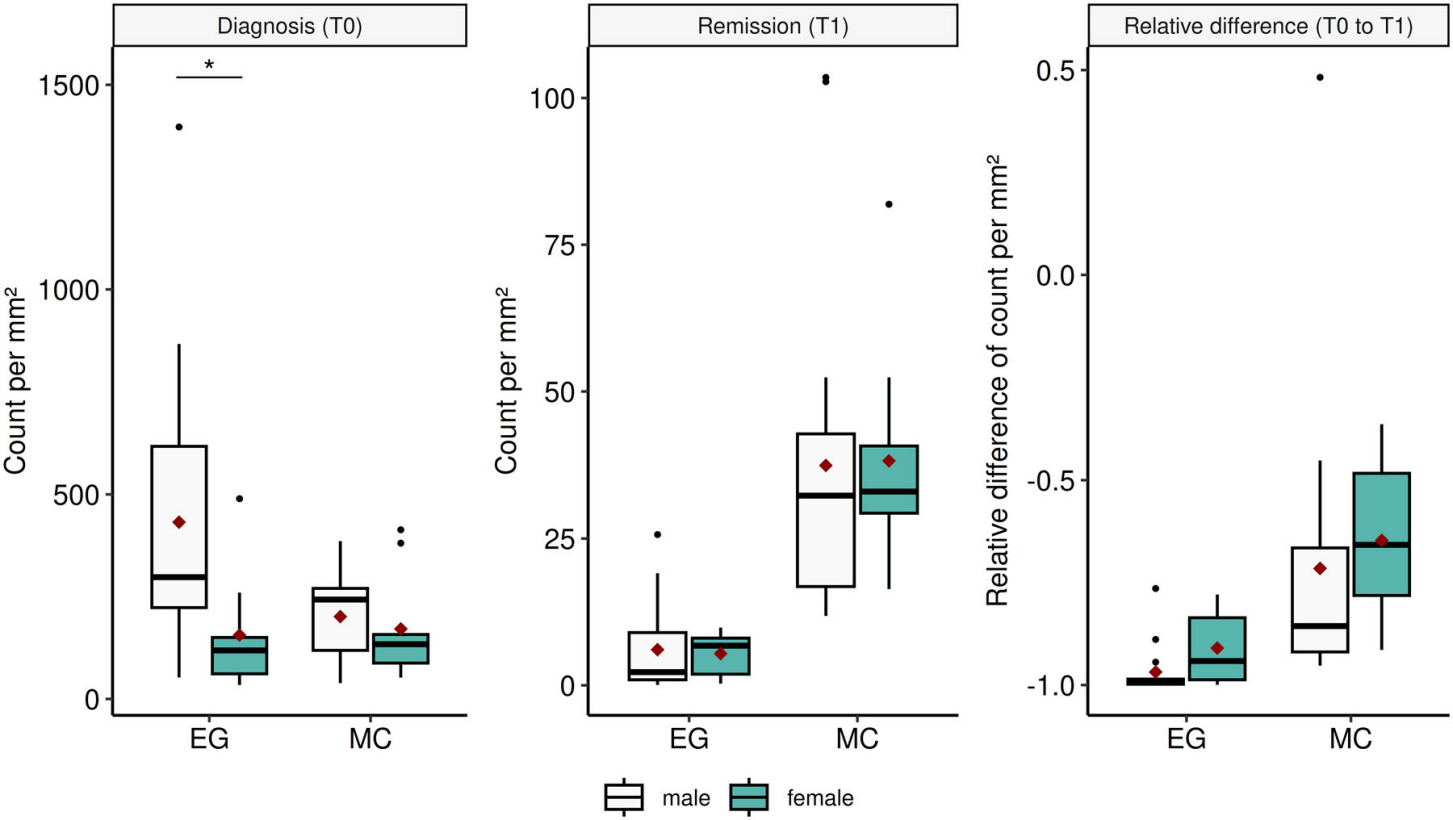
Cell counts and changes of gender within eosinophilic granulocytes (EG) and mast cells (MC). The diamonds represent the mean of each group. *p*‐Value: <0.001***, <0.01**, <0.05*.

**Figure 3:**
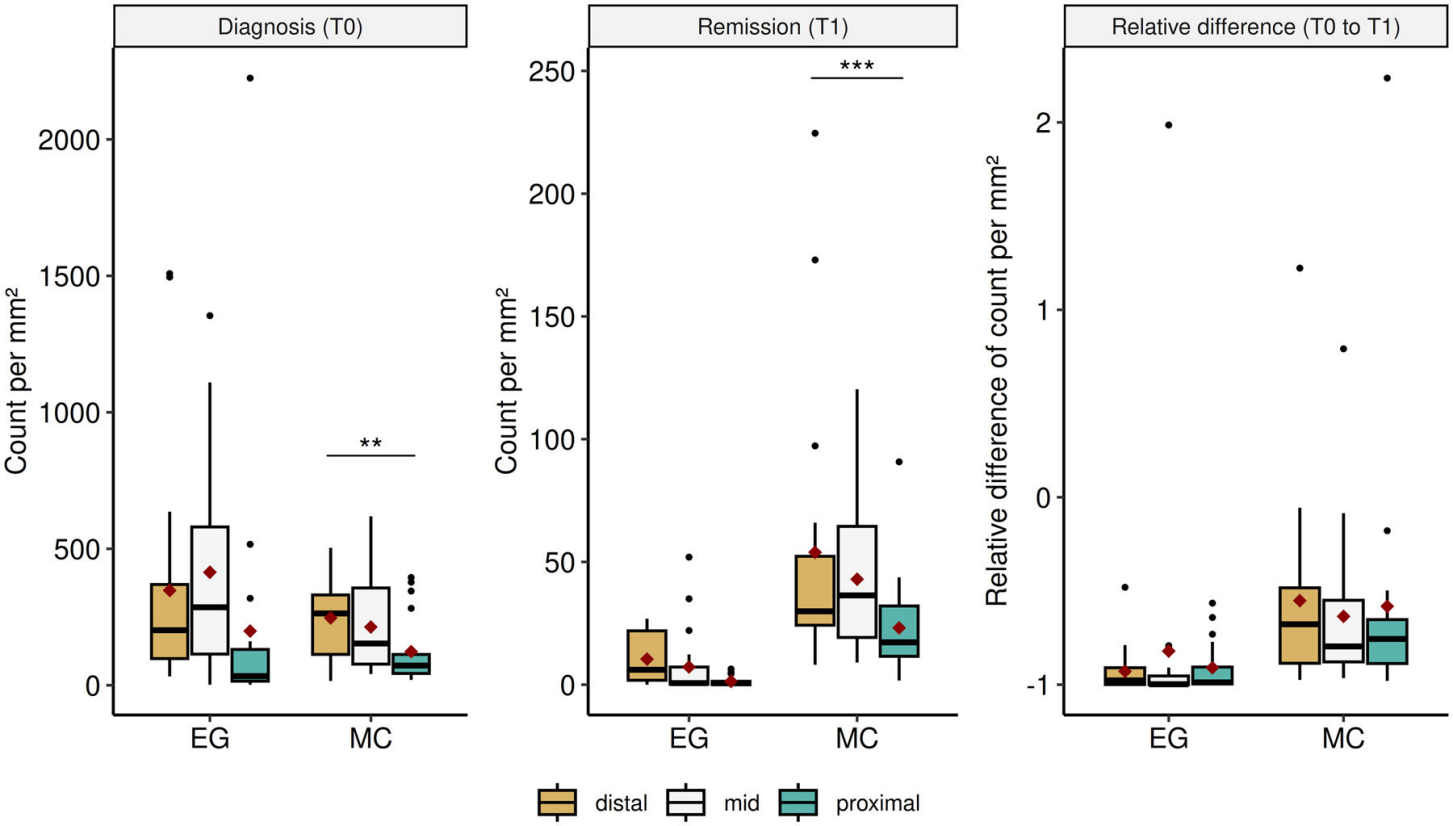
Cell counts and changes of the esophageal segments within eosinophilic granulocytes (EG) and mast cells (MC). The diamonds represent the mean of each group. *p*‐Value: <0.001***, <0.01**, <0.05*.

Next, we investigated whether the different cell subsets at the investigated time points differed within the subgroups namely type of therapy (PPI‐R vs. PPI‐NR), tissue type (squamous epithelium vs. other tissue), esophageal segments (proximal vs. mid vs. distal), gender (male vs. female) and relapse time (slow vs. fast) (Supporting Information: Table 2). We found a significant gender difference in esophageal eosinophilic granulocytes at diagnosis with boys displaying higher levels (male: median 297.66 [Q25‐Q75 223.24–617.40] vs. female: median 118.60 [Q25‐75 61.42–50.23], Supporting Information: Table 2A and Figure 2). Further, the esophageal mast cell count differed significantly among the different biopsy locations/esophageal segments at diagnosis (proximal: median 77.90 [Q25‐Q75 54.79–125.07] vs. mid: median 152.91 [Q25‐75 104.49–393.90] vs. distal: median 273.46 [Q25‐Q75 189.2–383.94]) as well as in histological remission (proximal: median 18.16 [Q25‐Q75 12.38–34.19] vs. mid: median 40.74 [Q25‐75 23.26–70.68] vs. distal: median 29.89 [Q25‐75 24.23–52.28]). While there seems to be an increase in MC infiltration from proximal to distal at diagnosis, in histological remission the highest cell count was found in the mid section (Supporting Information: Table 2B and Figure 3). Due to the small cohort size we were not able to perform post‐hoc tests to support this observation. All other comparisons yielded no significant results.

The comparison of cell counts within the subgroups of PPI‐R and PPI‐NR showed no significant differences. Despite not being significant, we observed that eosinophilic granulocytes at the time of diagnosis were higher in the group of PPI‐NR (median 268.64 [Q25‐75 187.62–349.60]) than in the PPI‐R (median 123.53 [Q25‐75 82.47–392.49]). At histological remission, however, the eosinophilic granulocytes cell count is higher in the PPI‐R than in the PPI‐NR (PPI‐NR: median 2.25 [Q25‐75 0.95–8.59] vs. PPI‐R: median 6.93 [Q25‐75 3.88–8.36]). Again, and as described above we found that the group of PPI‐NR displayed a higher MC tissue count than the PPI‐R at both time points analyzed (Supporting Information: Table 2B).

We also investigated the clinical symptoms and their possible association with the amount of esophageal MC infiltration in eosinophilic granulocyte‐based histological remission criteria. While dysphagia, reflux and heartburn were still present in cases with a higher MC count, the MC count was much lower and did not differ between patients without symptoms and those experiencing vomiting or abdominal pain (Supporting Information: Figure 1).

## 4 DISCUSSION

In this study, we aimed to analyze alterations of esophageal eosinophilic granulocytes and MC counts and alterations of cellular infiltrates over time from diagnosis to histological remission in pediatric EoE patients. We found that MC numbers decreased less and did not reach normal values even when histological remission was achieved by means of eosinophilic granulocytes <15/HPF. This is consistent with the findings of Bolton et al.,^2^ who recently discovered elevated levels of MC in pediatric patients with inactive EoE.

Of note, a discrepancy in eosinophilic granulocyte levels between male and female patients at the time of initial diagnosis was observed, with higher counts recorded in male patients. Further, a difference in between the esophageal segments for MC infiltrations both at diagnosis and at remission was examined with highest counts towards the middle and distal part of the esophagus at both time points.

In this study, we used the image analysis software MIKAIA® to facilitate the quantification of eosinophilic granulocytes and MC in EoE. MIKAIA v1.5.1 unmixes the components of the staining and then applies a computer vision‐based algorithm to detect cells. Using this method, it was possible to quickly analyze many digital sections. This required only a few adjustments to the basic parameter settings. However, a limitation is the lack of analysis of procedure time and subsequently cost efficiency. In general and in line with the trend of research on digital tools in pathology, it can be strongly assumed that with a high‐throughput volume of patient samples, the advantages will lie in the automated algorithm based analysis. Another limitation of this study is the time‐consuming and still necessary manual annotation, as unfortunately we did not yet succeed in establishing an independently AI‐supported and thus automated solution in defining ROI best by the end of the study. This includes the possibly associated lower precision and variation of manual annotations. On the other hand, however, it represents the current practice and standard in AI supported analyses of the GI‐tract.

As MIKAIA enables simplification of work, it may further gain in promoting the process of automation or implementation an AI‐algorithm. For example, Zhang et al. used machine learning for MC recognition in EoE, which enabled automation and the implementation of different features for detection and visualization. However, this also required a manual training and adaptation of the AI to achieve sufficient results and less error in detection.^12^ On the one hand, the current version of MIKAIA with manual annotation, slide scanning and software operation is time‐consuming and requires an expensive infrastructure. On the other hand, we are confident that it will allow robust and ultimately less expensive specialist use for specific applications in gastro‐pathology, such as EoE analysis in future. Challenges will lie in the optimal balance between the number of analyses and the variety of questions. Routine use will be possible, as we can already witness in many places today, e.g. Quantifier for PD‐L1, for Ki‐67 or hormone receptors in breast cancer, and so forth. The MIKAIA® software is a good example here, with several new updates for AI analysis already implemented at the time of this study's completion. These will address the challenges of our study and are likely to make future studies more efficient. Since currently there is no complete automation available either, our future research will focus on solving this issue.

The results of this study cohort demonstrated that in spite of a successful therapy resulting in eosinophilic granulocyte infiltrations below the widely accepted but arbitrarily chosen threshold of <15 cells/HPF, the number of MC remained increased.^2^ This may explain why certain symptoms such as dysphagia, reflux and heartburn still persist although histological remission was achieved thus emphasizing a potential role of MC in the pathophysiology of EoE. In addition, mast cells may contribute to esophageal edema formation, for example, by secreting histamine and leukotrienes. Furthermore, a sustained mast cell‐mediated inflammation may explain why earlier trials of IL‐5 blocker mepolizumab or benralizumab reduced eosinophil counts in the esophagus, but did not induce a significant improvement in symptoms.^13^, ^14^

It is known that the MC stimulate the activity of eosinophilic granulocytes,^3^ which is why they may be a target for therapies in the future.^9^ However, previous therapy attempts targeting MC using for example cromolyn did not achieve sufficient results possibly because of an insufficient pharmaceutical galenic.^15^

Further intense research on the pathophysiological involvement of MC in EoE and considering residual MC infiltrations under TCS‐therapy or IL4/IL13 blockade as well as the consideration as an additional therapeutic target could probably influence the course and treatment of EoE. The persistent elevated infiltration of MC during remission of EoE could explain why relief of clinical symptoms does sometimes not occur despite histological remission as defined per current criteria and why relapse is characteristic and very common in EoE.

Furthermore our study showed a difference of MC counts with respect to the biopsy position while we observed the highest counts in both in mid and distal localizations and lowest proximally. This observation may provide a direction in explaining why strictures in EoE are more pronounced distally—as already described in a study with mainly adult participants.^5^ However, this does not explain why the disease often presents clinically differently in pediatric patients than in adults, where the consequences of fibrosis resemble choking and bolus events as main symptoms. More research in this direction is still needed.

Moreover, it is known that male patients are more frequently affected by EoE or diagnosed with the disease.^7^, ^16^ Our data show that the number of eosinophilic granulocytes in male pediatric patients at the time of diagnosis was higher than in female patients. In the recently published study by Borinsky et al., male gender was identified among others as a clinical predictive factor for EoE in pediatric patients, to filter those affected to determine which should receive endoscopic examination.^17^ However, it is still unclear what causes the difference in clinical presentation between the sexes.^7^ In addition to the anatomical differences of the esophagus between male and female pediatric patients, histological differences must also be explored. To the best of our knowledge, we are the first to describe the gender difference in a pediatric cohort with regard to increased numbers of mucosal eosinophilic granulocytes and MC and their change in relation to histological remission after diagnosis.

Still, it would be important to investigate the gender difference further in depth. Furthermore, the observation of a tendency that eosinophilic granulocyte infiltration at the time of diagnosis was higher in the group of PPI‐NR than in the PPI‐R may provide a predictive hint for the right choice of therapy.

We are confident that a larger patient cohort in a future study would provide more precise or further findings in this regard and call for different therapeutic approaches.

## 5 CONCLUSION

In summary, MC seem to play an additional role in EoE pathophysiology as shown here and confirmed previously. In particular, the fact that MC remain elevated during histological remission calls for further research approaches possibly improving the therapy course by reducing events of frequent relapses. Despite the limitations of manual annotation, the automated analysis with MIKAIA® showed reproducible and robust results and routine cut‐offs, which make further studies on larger cohorts desirable to show a possible use in routine. Especially with constantly increasing numbers of examinations, robust and effective digital tools will be essential in future.

## CONFLICT OF INTEREST STATEMENT

The authors declare no conflicts of interest.

## Supporting information

The Supplementary.

The Supplementary.

The Supplementary.

The Supplementary.

The Supplementary.

The Supplementary.

The Supplementary.
