# Supplementary figures and images for: Mast cells infiltrates are common in eosinophilic esophagitis and still elevated in histological remission: A digital evaluation in children
Source: J Pediatr Gastroenterol Nutr. 2025 Jul 2;81(3):618–25. doi: 10.1002/jpn3.70137 (PMC12408972; doi:10.1002/jpn3.70137)

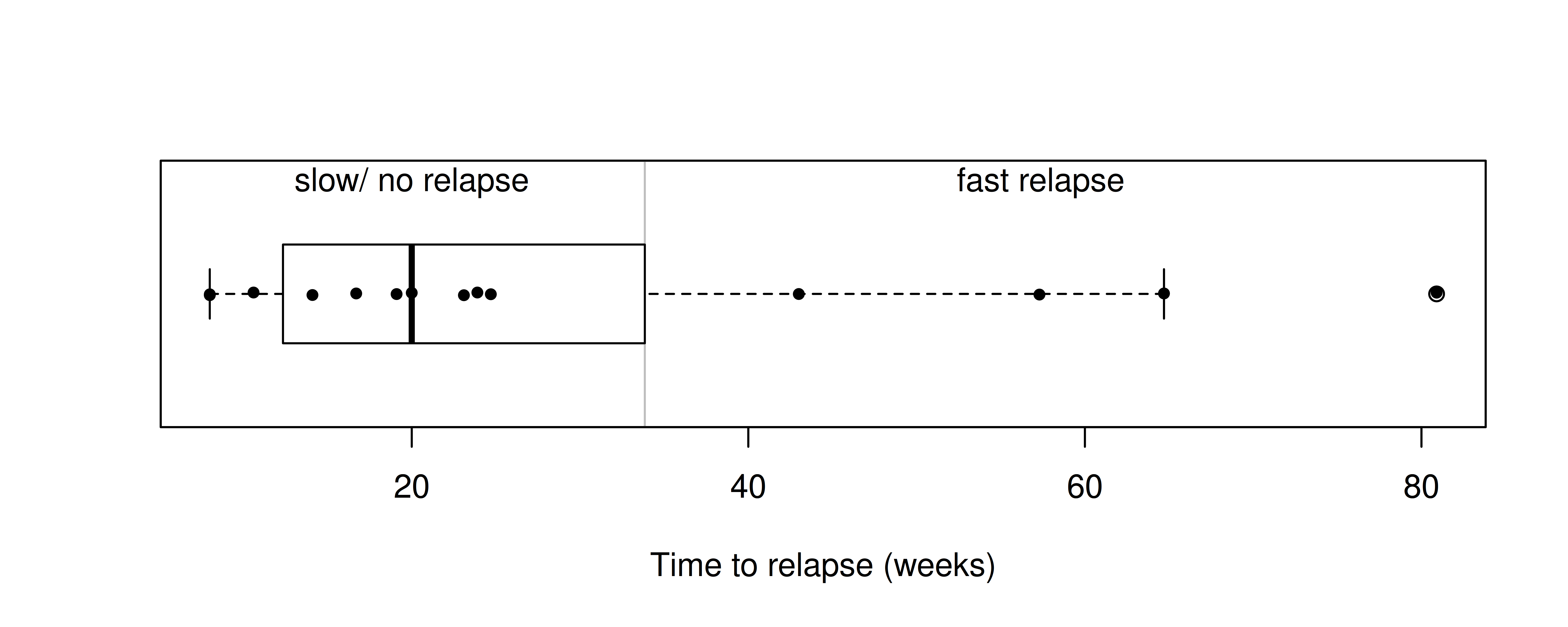

Supplement: Supplementary file 2 — The Supplementary. [file JPN3-81-618-s007.tiff]
