# Supplementary material for: Mast cells infiltrates are common in eosinophilic esophagitis and still elevated in histological remission: A digital evaluation in children
Source: J Pediatr Gastroenterol Nutr. 2025 Jul 2;81(3):618–25. doi: 10.1002/jpn3.70137 (PMC12408972; doi:10.1002/jpn3.70137)
Supplement: Supplementary file 3 — The Supplementary. [file JPN3-81-618-s002.pptx]

## Slide 1
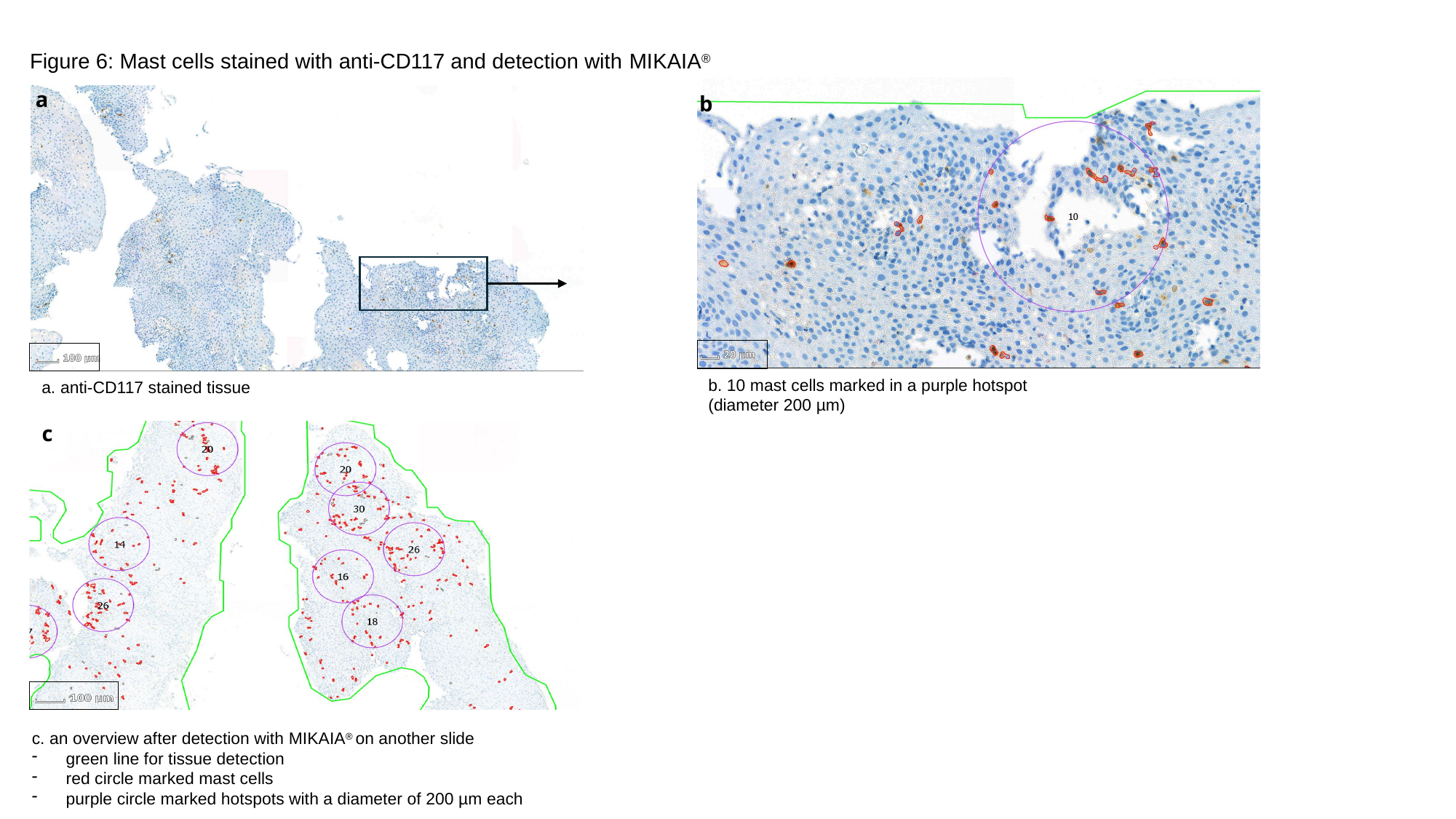

Figure 6: Mast cells stained with anti-CD117 and detection with MIKAIA®
a
b
b. 10 mast cells marked in a purple hotspot (diameter 200 µm)
a. anti-CD117 stained tissue
c
c. an overview after detection with MIKAIA® on another slide
green line for tissue detection
red circle marked mast cells
purple circle marked hotspots with a diameter of 200 µm each
