# Supplementary material for: Mast cells infiltrates are common in eosinophilic esophagitis and still elevated in histological remission: A digital evaluation in children
Source: J Pediatr Gastroenterol Nutr. 2025 Jul 2;81(3):618–25. doi: 10.1002/jpn3.70137 (PMC12408972; doi:10.1002/jpn3.70137)
Supplement: Supplementary file 4 — The Supplementary. [file JPN3-81-618-s001.pptx]

## Slide 1
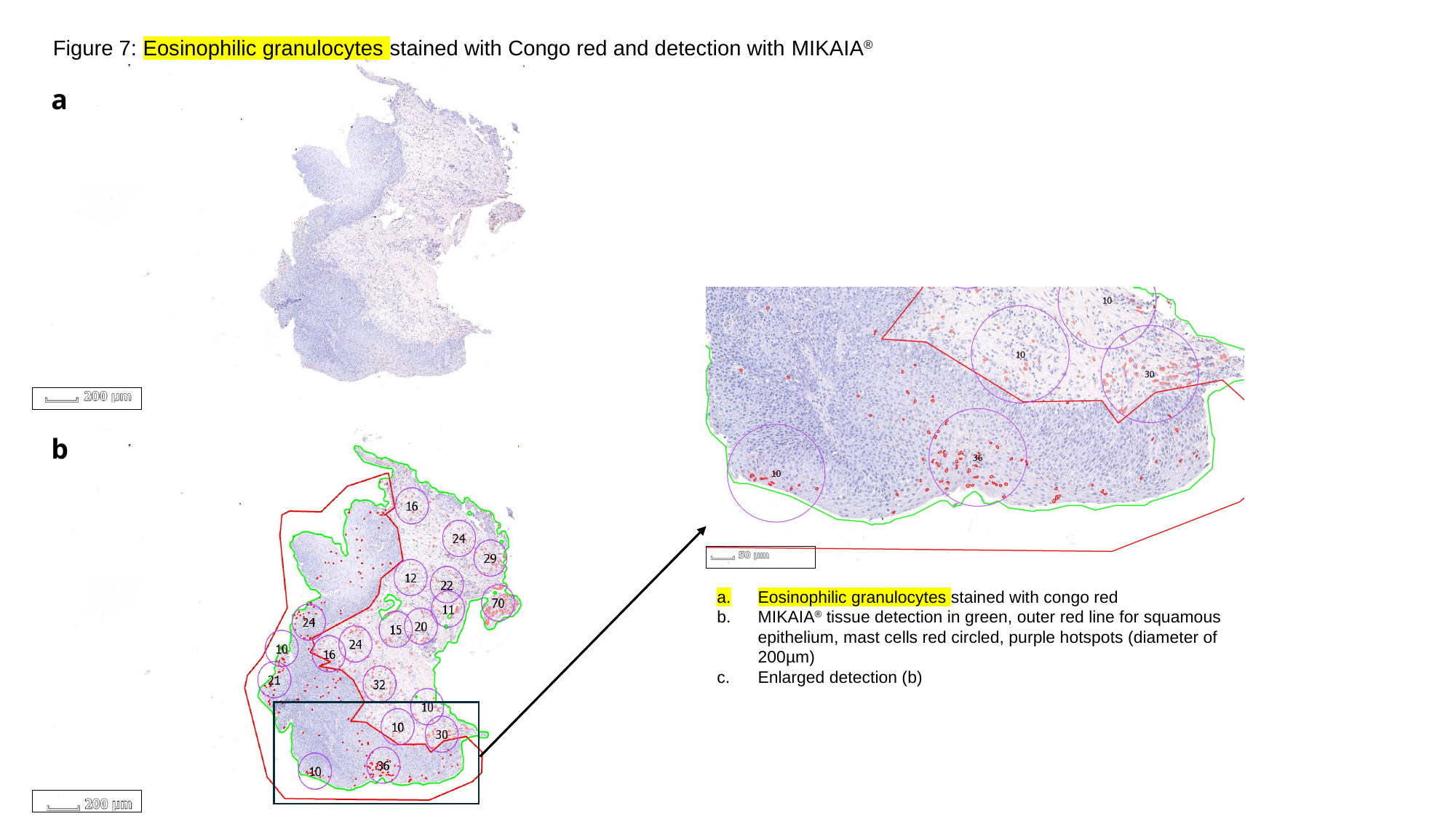

Figure 7: Eosinophilic granulocytes stained with Congo red and detection with MIKAIA®
a
c
b
Eosinophilic granulocytes stained with congo red
MIKAIA® tissue detection in green, outer red line for squamous epithelium, mast cells red circled, purple hotspots (diameter of 200µm)
Enlarged detection (b)
