# Supplementary material for: Mast cells infiltrates are common in eosinophilic esophagitis and still elevated in histological remission: A digital evaluation in children
Source: J Pediatr Gastroenterol Nutr. 2025 Jul 2;81(3):618–25. doi: 10.1002/jpn3.70137 (PMC12408972; doi:10.1002/jpn3.70137)
Supplement: Supplementary file 5 — The Supplementary. [file JPN3-81-618-s006.docx]

| **Eosinophilic granulocytes (EG) vs. Mast cells (MC)** | | | | | | | | | |
| --- | --- | --- | --- | --- | --- | --- | --- | --- | --- |
|  | **at diagnosis** | | | **at remission** | | | **relative difference** | | |
|  | **EG**  **Median**  **[Q25, Q75]** | **MC**  **Median**  **[Q25, Q75]** |  | **EG**  **Median**  **[Q25, Q75]** | **MC**  **Median**  **[Q25, Q75]** |  | **EG**  **mean**  **(sd)** | **MC**  **mean**  **(sd)** |  |
| \| **Cell type** \| \| --- \| | 245.42 | 160.08 |  | **2.38** | **32.67** | ******* | **-0.95** | **-0.69** | ******* |
|  | [114.91, 365.09] | [ 93.61, 265.38] |  | **[ 0.95, 8.59]** | **[ 18.49, 42.84]** |  | **(0.078)** | **(0.312)** |  |
| **Therapy** |  |  |  |  |  |  |  |  |  |
| PPI-R | 123.53 | 137.80 |  | 6.93 | 22.22 |  | **-0.93** | **-0.77** | ****** |
|  | [ 82.47, 392.49] | [ 62.14, 183.26] |  | [ 3.88, 8.36] | [ 19.05, 33.83] |  | **(0.082)** | **(0.112)** |  |
| PPI-NR | 268.64 | 203.81 |  | **2.25** | **35.44** | ****** | **-0.96** | **-0.65** | ****** |
|  | [187.62, 349.60] | [100.55, 283.42] |  | **[ 0.95, 8.59]** | **[ 24.69, 52.39]** |  | **0.076)** | **0.376)** |  |
| **Tissue type** |  |  |  |  |  |  |  |  |  |
| Squamous epithelium | 275.99 | 147.14 |  | **2.01** | **30.16** | ******* | **-0.97** | **-0.69** | ****** |
|  | [144.97, 535.93] | [ 89.51, 275.34] |  | **[ 0.52, 8.87]** | **[ 17.67, 35.69]** |  | **(0.061)** | **(0.328)** |  |
| other | 118.60 | 133.91 |  | **0.00** | **39.50** | ****** | -1.00 | -0.46 |  |
|  | [ 34.20, 236.98] | [ 72.19, 174.18] |  | **[ 0.00, 3.05]** | **[ 0.00, 132.47]** |  | (0.013) | (0.685) |  |
| **Esophageal segments** |  |  |  |  |  |  |  |  |  |
| proximal | 33.48 | 72.05 |  | **0.59** | **17.25** | ******* | -0.93 | -0.55 |  |
|  | [ 14.94, 131.00] | [ 43.24, 112.59] |  | **[ 0.00, 1.36]** | **[ 11.55, 32.05]** |  | (0.128) | (0.528) |  |
| mid | 285.62 | 152.87 |  | **0.64** | **36.37** | ******* | -0.82 | -0.64 |  |
|  | [114.09, 579.82] | [ 77.38, 356.40] |  | **[ 0.21, 7.18]** | **[ 19.24, 64.47]** |  | (0.647) | (0.413) |  |
| distal | 201.50 | 263.03 |  | **6.07** | **29.89** | ******* | -0.91 | -0.58 |  |
|  | [ 97.61, 369.05] | [113.04, 330.75] |  | **[ 1.80, 21.93]** | **[ 24.23, 52.28]** |  | (0.141) | (0.752) |  |
| **Gender** |  |  |  |  |  |  |  |  |  |
| male | 297.66 | 242.93 |  | **2.25** | **32.32** | ******* | -0.97 | -0.72 |  |
|  | [223.24, 617.40] | [118.70, 270.19] |  | **[ 0.94, 8.98]** | **[ 16.83, 42.84]** |  | (0.064) | (0.363) |  |
| female | 118.60 | 133.80 |  | **6.75** | **33.02** |  | **-0.91** | **-0.65** | ****** |
|  | [ 61.42, 150.23] | [ 87.65, 157.50] |  | **[ 1.91, 8.04]** | **[ 29.33, 40.77]** |  | **(0.092)** | **(0.197)** |  |
| **Relapse** |  |  |  |  |  |  |  |  |  |
| slow/no relapse | 277.31 | 164.68 |  | **2.25** | **23.39** | ******* | **-0.97** | **-0.80** | ******* |
|  | [143.22, 323.62] | [133.80, 260.56] |  | **[ 0.48, 6.17]** | **[ 17.06, 35.44]** |  | **(0.061)** | **(0.143)** |  |
| fast relapse | 230.86 | 141.80 |  | **8.20** | **34.78** | ****** | -0.92 | -0.55 |  |
|  | [ 90.01, 393.16] | [ 78.74, 274.16] |  | **[ 1.91, 11.75]** | **[ 32.36, 51.39]** |  | (0.093) | (0.415) |  |

Note: <0.001***, <0.01**, <0.05*
