# Supplementary material for: Mast cells infiltrates are common in eosinophilic esophagitis and still elevated in histological remission: A digital evaluation in children
Source: J Pediatr Gastroenterol Nutr. 2025 Jul 2;81(3):618–25. doi: 10.1002/jpn3.70137 (PMC12408972; doi:10.1002/jpn3.70137)
Supplement: Supplementary file 6 — The Supplementary. [file JPN3-81-618-s005.docx]

| ***A) Eosinophilic granulocytes*** |  |  |  |
| --- | --- | --- | --- |
|  | **at diagnosis**  **(Median [Q25, Q75], Wilcoxon test)** | **at remission**  **(Median [Q25, Q75], Wilcoxon test)** | **relative difference**  **(Mean (sd), t-Test)** |
| **Gender** |  |  |  |
| male vs. female | **297.66 vs. 118.60***  **[223.24, 617.40] [ 61.42, 150.23]** | 2.25 vs. 6.75  [ 0.94, 8.98] [ 1.91, 8.04] | 6.07 vs. 5.41  ( 7.720) ( 3.817) |
| **Esophageal segments** |  |  |  |
| proximal vs. mid vs. distal^1^ | 35.65 vs. 288.22 vs. 183.14  [ 15.53, 145.62] [113.05, 582.85] [ 95.97, 343.46] | 0.77 vs. 1.14 vs. 6.07  [ 0.14, 1.36] [ 0.24, 7.18] [ 1.80, 21.93] | -0.92 vs. -0.72 vs. -0.89  ( 0.147) ( 0.818) ( 0.154) |
| **Relapse** |  |  |  |
| slow/no relapse vs. fast relapse | 277.31 vs. 230.86  [143.22, 323.62] [ 90.01, 393.16] | 2.25 vs. 8.20  [ 0.48, 6.17] [ 1.91, 11.75] | 3.54 vs. 8.83  ( 3.424) ( 8.429) |
| **Therapy** |  |  |  |
| PPI-NR vs. PPI-R | 268.64 vs. 123.53  [187.62, 349.60] [ 82.47, 392.49] | 2.25 vs. 6.93  [ 0.95, 8.59] [ 3.88, 8.36] | 5.68 vs. 6.14  ( 7.631) ( 4.180) |
| **Tissue type** |  |  |  |
| Squamous epithelium vs. other | 293.08 vs. 126.68  [124.06, 522.41] [ 32.93, 285.36] | 2.25 vs. 0.00  [ 0.52, 9.06] [ 0.00, 1.52] | -0.96 vs. -1.00  ( 0.064) ( 0.014) |
| ***B) Mast cells*** |  |  |  |
|  | **at diagnosis**  **(Median [Q25, Q75], Wilcoxon test)** | **at remission**  **(Median [Q25, Q75], Wilcoxon test)** | **relative difference**  **(Mean (sd), t-Test)** |
| **Gender** |  |  |  |
| male vs. female | 242.93 vs. 133.80  [118.70, 270.19] [ 87.65, 157.50] | 32.32 vs. 33.02  [ 16.83, 42.84] [ 29.33, 40.77] | 37.47 vs. 38.25  ( 29.573) ( 20.731) |
| **Esophageal segments** |  |  |  |
| proximal vs. mid vs. distal^1^ | **77.90 vs. 152.91 vs. 273.46 ****  **[ 54.79, 125.07] [104.49, 393.90] [189.26, 383.94]** | **18.16 vs. 40.74 vs. 29.89 *****  **[ 12.38, 34.19] [ 23.26, 70.68] [ 24.23, 52.28]** | -0.54 vs. -0.57 vs. -0.54  ( 0.606) ( 0.504) ( 0.860) |
| **Relapse** |  |  |  |
| slow/no relapse vs. fast relapse | 164.68 vs. 141.80  [133.80, 260.56] [ 78.74, 274.16] | 23.39 vs. 34.78  [ 17.06, 35.44] [ 32.36, 51.39] | 31.88 vs. 45.35  ( 24.529) ( 27.909) |
| **Therapy** |  |  |  |
| PPI-NR vs. PPI-R | 203.81 vs. 137.80  [100.55, 283.42] [ 62.14, 183.26] | 35.44 vs. 22.22  [ 24.69, 52.39] [ 19.05, 33.83] | 43.84 vs. 26.31  ( 30.092) ( 11.807) |
| **Tissue type** |  |  |  |
| Squamous epithelium vs. other | 157.59 vs. 112.16  [ 95.47, 306.04] [ 49.17, 155.42] | 30.81 vs. 32.96  [ 20.48, 41.63] [ 0.00, 123.05] | -0.65 vs. -0.42  ( 0.370) ( 0.693) |
| Note: <0.001***, <0.01**, <0.05*  ^1^ Tests used for threeway comparison: Friedman, Friedman, ANOVA | | | |
