# Supplementary material for: Mast cells infiltrates are common in eosinophilic esophagitis and still elevated in histological remission: A digital evaluation in children
Source: J Pediatr Gastroenterol Nutr. 2025 Jul 2;81(3):618–25. doi: 10.1002/jpn3.70137 (PMC12408972; doi:10.1002/jpn3.70137)
Supplement: Supplementary file 7 — The Supplementary. [file JPN3-81-618-s004.docx]

**Supplemental Information MIKAIA®.**

First, after importing the slides into the software, a tissue detection was carried out using suitable parameters that outlines tissue particles and therefore separates foreground from background. Any detected foreground not suited for analysis, such as debris were then manually removed from the detected foreground mask. Next, the IHC Cell Detection App was used to detect and outline cells and grade them into positive or negative based on their mean 3,3'-Diaminobenzidin (DAB) intensity. The app’s computer vision-based algorithm was selected, which first unmixes the two stains using a color deconvolution and then detects cells using a combination of preprocessing and thresholding steps, morphological operations and filtering based on morphological and color attributes. For IHC, we selected MIKAIA’s “H-DAB” stain unmixing preset and for Congo red, the software’s built in stain estimation feature was used to obtain a custom stain unmixing matrix. The cell detection app’s optional built-in hotspot search was configured to provide the top 10 circular hotspots of 200 µm diameter. The squamous epithelial annotations were fed into the IHC Cell Detection module as a region of interest so that it automatically groups any detected cells into inside vs. outside the squamous epithelium and reports statistics such as cell abundance, density in cells/mm² or detected hotspots individually per region. After preliminary cell detection runs, any remaining difficult regions that continued to yield false positive cell detections despite careful parameter selection were manually marked (“ignore” annotation class) and this way detections inside these regions were then automatically discarded in the final analysis. Initially, a senior pathologist validated the obtained image analysis results on a test set. This step was preceded by a period of meticulous and exhaustive research, during which a variety of automated annotation approaches were evaluated. Our goal at this stage of the project was to achieve a comprehensive, automated, and observer-independent workflow. Two distinct neuronal networks (based on RAS-Net and Alex-Net, supervised learning, CNN) were unable to adequately separate epithelia (epithelium and underlying tissue were separated ROIs). A further approach using an artificial intelligence (AI) from a third-party also failed to achieve satisfactory results using available training data. Hence in this respect, none of the previous AI solutions were able to perform acceptably. To circumvent the stagnation of the evaluation process, a semi-automatic approach was employed, augmented by manual annotations, thereby enabling subsequent evaluation with MIKAIA®. Then, the entire cohort was batch-analyzed using the same image analysis parameter settings. The software enabled a completely digital evaluation of the cells and cell count independent of the individual examiner.
